# Bcl-2/Bax protein ratio predicts 5-fluorouracil sensitivity independently of p53 status

**DOI:** 10.1054/bjoc.2000.1455

**Published:** 2000-10-26

**Authors:** J-F Mirjolet, M Barberi-Heyob, C Didelot, J-P Peyrat, J Abecassis, R Millon, J-L Merlin

**Affiliations:** Centre Alexis Vautrin, Laboratoire de Recherche en Oncologie, Avenue de Bourgogne, Vandœuvre-les-Nancy cedex, 54511; Centre Oscar Lambret, Laboratoire d'Oncologie Moléculaire Humaine, Lille cedex, 59020; Centre Paul Strauss, Laboratoire de Biologie Tumorale, 3 rue de la Porte de l'Hôpital, Strasbourg cedex, 67085, France

**Keywords:** 5-FU sensitivity, p53 status, mdm2, G1/S arrest, Bcl-2/Bax ratio

## Abstract

p53 tumour-suppressor gene is involved in cell growth control, arrest and apoptosis. Nevertheless cell cycle arrest and apoptosis induction can be observed in p53-defective cells after exposure to DNA-damaging agents such as 5-fluorouracil (5-FU) suggesting the importance of alternative pathways via p53-independent mechanisms. In order to establish relationship between p53 status, cell cycle arrest, Bcl-2/Bax regulation and 5-FU sensitivity, we examined p53 mRNA and protein expression and p53 protein functionality in wild-type (wt) and mutant (mt) p53 cell lines. p53 mRNA and p53 protein expression were determined before and after exposure to equitoxic 5-FU concentration in six human carcinoma cell lines differing in p53 status and displaying marked differences in 5-FU sensitivity, with IC _50_ values ranging from 0.2–22.6 mM. 5-FU induced a rise in p53 mRNA expression in mt p53 cell lines and in human papilloma virus positive wt p53 cell line, whereas significant decrease in p53 mRNA expression was found in wt p53 cell line. Whatever p53 status, 5-FU altered p53 transcriptional and translational regulation leading to up-regulation of p53 protein. In relation with p53 functionality, but independently of p53 mutational status, after exposure to 5-FU equitoxic concentration, all cell lines were able to arrest in G1. No relationship was evidenced between G1 accumulation ability and 5-FU sensitivity. Moreover, after 5-FU exposure, Bax and Bcl-2 proteins regulation was under p53 protein control and a statistically significant relationship (*r*= 0.880,*P*= 0.0097) was observed between Bcl-2/Bax ratio and 5-FU sensitivity. In conclusion, whatever p53 status, Bcl-2 or Bax induction and Bcl-2/Bax protein ratio were correlated to 5-FU sensitivity. © 2000 Cancer Research Campaign


*British Journal of Cancer* (2000) **83**(10), 1380–1386
© 2000 Cancer Research Campaign
doi: 10.1054/ bjoc.2000.1455, available online at http://www.idealibrary.com on IDEAL®

### Bcl-2/Bax protein ratio predicts 5-fluorouracil sensitivity independently of p53 status

**J-F Mirjolet, M Barberi-Heyob[1], C Didelot[1], J-P Peyrat[2], J Abecassis[3], R Millon[3] and J-L Merlin[1]**

[1]Centre Alexis Vautrin, Laboratoire de Recherche en Oncologie, Avenue de Bourgogne, 54511 Vandœuvre-les-Nancy cedex; [2]Centre Oscar Lambret, Laboratoire d'Oncologie Moléculaire Humaine, 59020 Lille cedex; [3]Centre Paul Strauss, Laboratoire de Biologie Tumorale, 3, rue de la Porte de l'Hôpital, 67085 Strasbourg cedex, France

**Summary** p53 tumour-suppressor gene is involved in cell growth control, arrest and apoptosis. Nevertheless cell cycle arrest and apoptosis induction can be observed in p53-defective cells after exposure to DNA-damaging agents such as 5-fluorouracil (5-FU) suggesting the importance of alternative pathways via p53-independent mechanisms. In order to establish relationship between p53 status, cell cycle arrest, Bcl-2/Bax regulation and 5-FU sensitivity, we examined p53 mRNA and protein expression and p53 protein functionality in wild-type (wt) and mutant (mt) p53 cell lines. p53 mRNA and p53 protein expression were determined before and after exposure to equitoxic 5-FU concentration in six human carcinoma cell lines differing in p53 status and displaying marked differences in 5-FU sensitivity, with $IC_{50}$ values ranging from 0.2–22.6 mM. 5-FU induced a rise in p53 mRNA expression in mt p53 cell lines and in human papilloma virus positive wt p53 cell line, whereas significant decrease in p53 mRNA expression was found in wt p53 cell line. Whatever p53 status, 5-FU altered p53 transcriptional and translational regulation leading to up-regulation of p53 protein. In relation with p53 functionality, but independently of p53 mutational status, after exposure to 5-FU equitoxic concentration, all cell lines were able to arrest in G1. No relationship was evidenced between G1 accumulation ability and 5-FU sensitivity. Moreover, after 5-FU exposure, Bax and Bcl-2 proteins regulation was under p53 protein control and a statistically significant relationship ($r$ = 0.880, $P$ = 0.0097) was observed between Bcl-2/Bax ratio and 5-FU sensitivity. In conclusion, whatever p53 status, Bcl-2 or Bax induction and Bcl-2/Bax protein ratio were correlated to 5-FU sensitivity. © 2000 Cancer Research Campaign

**Keywords**: 5-FU sensitivity; p53 status; mdm2; G1/S arrest; Bcl-2/Bax ratio p53 tumour-suppressor gene is involved in the control of cell growth, arrest and apoptosis (Kastan et al, 1991; Oren, 1994; Stewart et al, 1995). Cells exposed to DNA-damaging agents such as 5-fluorouracil (5-FU) activate wild-type p53, then either undergo G1/S arrest and are repaired or undergo apoptosis (Kastan et al, 1991; Kuerbitz et al, 1992; Guillouf et al, 1995). The option which prevails may be reflected by the relative levels of $p21^{WAF1}$ and/or Bcl-2 gene family expression. However, apoptosis induction has also been reported in p53-defective cells after exposure to DNA-damaging agents, suggesting the importance of alternative pathways (Dou et al, 1995). As currently accepted, biosynthesis of wild-type p53 can be controlled by both transcriptional (Deffie et al, 1993; Hudson et al, 1995) and translational regulation processes (Mosner et al, 1995; Ewen and Miller, 1996; Fu et al, 1996). Inhibition of p53 biosynthesis by translational process requires wild-type p53 and arises through a negative autoregulatory feedback loop (Mosner et al, 1995; Ewen and Miller, 1996; Fu et al, 1996). Although the precise mechanism through which transcriptional autoregulation is mediated still remains to be elucidated, this effect appears to be cell-type specific and to involve binding of p53 to other transcription factors. It has now been reported that mutation as well as other factors can stabilize p53 protein and render it non-functional (Vogelstein and Kinzler, 1992). Interactions with viral or cellular proteins such as HPV-E6 protein or mdm2 gene product (Scheffner et al, 1990; Kubbutat et al, 1997) were shown to inactivate p53 protein. p53 and mdm2 proteins constitute an autoregulatory feedback loop in which p53 limits its own activity through the production of mdm2 (Momand et al, 1992; Barak et al, 1993). These observations have now led to the theory that the whole cellular environment may determine p53 stability and function. These data suggest that stabilized detectable p53 protein, whether created as a result of mutation or by some other protein interaction, may have inactivated or impaired function in the cell, such as apoptosis induction.

Chemotherapeutic agents including 5-FU are known to induce apoptosis (Lowe et al, 1993). Differences in p53 functionality between cell lines displaying different 5-FU sensitivity could result from p53 functional status inducing various cellular responses to drug-induced damage. Bcl-2 belongs to a growing family of apoptosis regulators and experiments suggested the involvement of p53 and Bcl-2 family proteins in chemotherapy-induced apoptosis (Harris, 1996; Nita et al, 1998*a*). Bcl-2 and Bcl-xl can block cell death in various cell systems under a variety of conditions. Conversely, overexpression of Bax, Bak and Bad, among the other Bcl-2 family proteins, was shown to induce apoptosis (Strobel et al, 1996).

Nita et al (1998*a*) have recently demonstrated that, in cells expressing wild-type p53, 5-FU-induced apoptosis was accompanied by increased expression of Bax and Bak without consistent modulation of other Bcl-2 family proteins as opposed to cells

*Received 3 May 2000*
*Revised 18 July 2000*
*Accepted 18 July 2000*

*Correspondence to:* M Barberi-Heyob containing mutant-type p53. In the present study, we investigated the difference in 5-FU sensitivity observed in six human cancer cell lines with different p53 status and tried to evidence the basis of this difference by following Bcl-2/Bax ratio and its correlation with p53 status and mRNA or protein expression induction after exposure to 5-FU equitoxic concentration. Whether the 5-FU sensitivity differences are a result of different p53 functionality causing differences in Bcl-2 and Bax regulation, the implication of p53 protein expression induction in G1 arrest ability will be discussed and reconsidered.

### MATERIALS AND METHODS

### Materials and chemicals

Cell culture materials were purchased from Costar (Dutscher, Brumath, France), culture media and additives from Life Technologies (Gibco BRL, Cergy-Pontoise, France), except for fetal calf serum, which was obtained from Costar. Taq-polymerase, RNAse H, random primers, SuperScript II® DNA polymerase, deoxynucleotide triphosphate were purchased from Life Technologies. Anti-bromodeoxyuridin monoclonal antibodies, p53 monoclonal antibodies (DO-7) and peroxidase-conjugated antibodies were provided by Dako (Trappes, France). Bax (N-20) polyclonal antibodies were purchased from Tebu (Le Perray-en-Yvelines, France). All other chemicals were purchased from Sigma (St Quentin Fallavier, France) and were of molecular biology grade.

### Cell culture

CAL51 human breast adenocarcinoma, PANC3 pancreas carcinoma, CAL27 and CAL33 human head and neck carcinoma cell lines were kindly provided by Dr JL Fischel (Centre Antoine Lacassagne, Nice, France). FaDu and KB, head and neck carcinoma cell lines, were obtained from Professor A Hanauske (Munich University, Germany) as part of the EORTC Preclinical Therapeutic Models Group exchange program. All cell lines were grown in 75 $cm^2$ plastic tissue culture flasks in RPMI 1640 medium supplemented with 10% heat inactivated fetal calf serum, penicillin (100 iu $ml^{-1}$), streptomycin (100 µg $ml^{-1}$) in a 37°C, 5% $CO_2$ atmosphere. The cells were exposed at day 4 after seeding to equitoxic 5-FU concentrations ($IC_{50}$) for 24 h, then analysed immediately.

### Cytotoxicity assay

MTT assays were carried out according to a procedure previously reported (Barberi-Heyob et al, 1993). Briefly, cells were seeded at the initial density of $2.10^4$ cells $ml^{-1}$ in 96-well micro titration plates. 72 h after plating, cells were exposed for 72 h to 5-FU concentrations ranging from 0.08–$4.10^4$ µM, each concentration being tested in sextuplicate. 50 µl of 0.5% MTT solution were then added in each well and incubated for 3 h at 37°C to allow MTT metabolization. The formazan crystals were dissolved by adding 50 µl per well of 25% sodium dodecylsulfate solution and vigorous pipetting. Absorbance was measured at 540 nm using a Multiskan MCC/340 plate reader (Labsystem, Cergy-Pontoise, France). Results were expressed as relative absorbance to untreated controls. 5-FU concentrations yielding 50% growth inhibition ($IC_{50}$) were calculated using medium effect algorithm (Chou and Talalay, 1987) and expressed as mean values of five independent experiments.

### Analysis of p53 mutations

To identify p53 genomic mutation, direct DNA automated fluorescent sequencing analyses were conducted. Both DNA strands were sequenced. Briefly, PCR was performed using four pairs of primers covering exons 2–9 and including flanking intronic splicing sites (one pair for exons 2–4, one for exons 5 and 6, one for exon 7 and finally one pair for exons 8 and 9), in a 20 µl volume containing 10 mmol $l^{-1}$ Tris-HCl, 50 mmol $l^{-1}$ KCl, 1.5 mmol $l^{-1}$ $MgCl_2$, 0.2 mmol $l^{-1}$ deoxynucleotide triphosphates, 0.5 µmol $l^{-1}$ of each primer, and 1 µg of genomic DNA. The reactions were carried out using a Perkin Elmer/Cetus thermal cycler model 9600. The PCR products were then purified using Sephacryl S400HR (Amersham-Pharmacia Biotech, Les Ulis, France). 5 µl of purified fragments were used for sequencing with a Thermo Sequenase™ Dye Terminator Cycle Sequencing kit (Amersham-Pharmacia Biotech), using the same PCR primers. After purification with Biogel P10 (Bio Rad), the products were sequenced using ABI 373 automated DNA sequencing system (Applied Biosystem).

### RNA isolation and RT-PCR analysis

Isolation of total RNA was performed using TRIzol® according to the manufacturer's specifications (Life Technologies). cDNA synthesis was performed with 1 µg total RNA in a reaction volume of 20 µl containing 100 ng of random primers, 50 mM Tris-HCl, pH 8.3, 75 mM KCl, 3 mM $MgCl_2$, 0.5 mM deoxynucleotide triphosphate, 10 mM dithiothreitol and 200 units SuperScript II® reverse transcriptase and incubated for 10 min at room temperature, 50 min at 42°C, followed by 15 min at 70°C. RNAse-H (2.5 units) was added into each sample, then incubated for 20 min at 37°C. cDNA samples were stored at –20°C until analysed.

#### *p53 and p21*

p53 and p21 semi-quantitative PCR analyses were then performed using β2-microglobulin ($\beta_2$m) as reference gene. 0.5 µl or 1 µl of cDNA samples were mixed, respectively for p53 or p21 amplification, in a volume of 20 µl containing 16 mM $(NH_4)_2SO_4$, 67 mM Tris-HCl, pH 8.8, 0.01% Tween 20, 2 or 1.5 mM $MgCl_2$ respectively for p53 or p21 amplification, 0.2 mM dNTP, 5 µM of each 5′- and 3′-primers, and 0.5 unit of Taq polymerase. The primers sequences were 5′-TCTGTGACTTGCACGTACTC-3′ (sense) and 5′-CACGGATCTGAAGGGTGAAA-3′ (antisense) for p53 (Aguilar Santelises et al, 1996), 5′-CCCAGTGGACAGCGAGCAGC-3′ (sense) and 5′-ACTGCAGGCTTCCTGTGGGC-3′ (antisense) for p21, 5′-ACCCCCACTGAAAAAGATGA-3′ (sense) and 5′-ATCTTCAAACCTCCATGATG-3′ (antisense) for $\beta_2$-microglobulin ($\beta_2$m) (Gussow et al, 1987).

The PCR tubes were incubated for p53 and $\beta_2$m amplification, as follows: the first cycle was 5 min at 95°C, 1 min at 57°C and 1 min at 72°C. The 33 or 36 following cycles, respectively for p53 and p21 amplification, were 1 min at 94°C, 1 min at 57°C and 1 min at 72°C. In each case, after completion of PCR cycles, the mixture was finally incubated for 7 min at 72°C. p53 and $\beta_2$ PCR products were electrophoretized on 1% agarose gel containing 0.1 µg $ml^{-1}$ of ethidium bromide. Quantification was performed by UV transillumination using a Gel Doc 1000 system (Bio Rad,

© 2000 Cancer Research Campaign

**Table 1** Characteristics of the cell lines used. Identification of p53 genomic mutations covering exons 2–9 and 5-FU sensitivity ($IC_{50}$)

| Cell lines | Exon | Codon | Nucleoside substitution | Amino-acid substitution[a] | p53 status[b] | 5-FU $IC_{50}$[c] (mM) |
|---|---|---|---|---|---|---|
| CAL51 | 4 | 72 | CGC → CCC | R → P | wt | 0.16 ± 0.01 |
| KB | – | – | – | – | wt | 0.94 ± 0.17 |
| FaDu | 7 | 248 | CGG → CTG | R → L | mt | 1.05 ± 0.48 |
| | 7 | splicing site | TAG → TAA | – | – | – |
| CAL33 | 4 | 72 | CGC → CCC | R → P | mt | 0.97 ± 0.63 |
| | 5 | 175 | CGC → CAC | R → H | – | – |
| CAL27 | 4 | 72 | CGC → CCC | R → P | mt | 3.12 ± 0.84 |
| | 6 | 193 | ATC → TTC | H → L | – | – |
| PANC3 | 2–4 | – | deletion | – | del | 22.62 ± 3.09 |

[a] R = arginine; P = proline; L = leucine; H = histidine; [b]mt = mutant-type; wt = wild-type; del = deletion; [c]mean ± standard deviation of five independent experiments

Ivry-sur-Seine, France). Finally, for each cDNA sample, p53: $\beta_2$m relative expression ratio (RER) was calculated as the ratio of the fluorescence intensities of p53 and $\beta_2$m PCR products bands.

### *Mdm2*

Mdm2 gene contains two different promoter regions. The upstream promoter region (P1) is known to be active in absence of p53 and the second promoter region (P2) is located within the first intron and contains a p53-responsive element (mdm2-p53RE).

The multiPCR of these different transcripts was performed using the forward mdm2 exon1-specific and mdm2 exon2-specific primers (5′-GAAAAGATGGAGCAAGAAGCC-3′ and 5′-CAG-TGGC-GATTGGAGGGTAG-3′), respectively with a unique reverse primer (5′-GTAGGTACAGACATGTTGGTA-3′) located in exon3 of the mdm2 gene. Amplification of $\beta_2$m was performed concomitantly using the forward (5′-AGCAGAGAATGGAA-AGTCAAA-3′) and reverse (5′-TGTTGATGTTGGATAAGA-GAAT-3′) primers. The reaction volume was 50 µl and comprised 1X reaction buffer, 1.5 mM $MgCl_2$, 0.2 µM of each mdm2 forward primers, 0.4 µM of mdm2 reverse primer and 0.05 µM of $\beta_2$m primers, 0.25 mM of deoxynucleotides and 1 unit of HotStarTaq DNA polymerase (Qiagen, Courtaboeuf, France). Amplification was carried out for 30 cycles of 94°C for 1 min, 55°C for 30 s and 72°C for 30 s using a thermal cycler (Perkin Elmer 480). The cycles were followed by incubation of the mixtures for 15 min at 95°C to ensure full denaturation of the target DNA and activation of HotStarTaq DNA polymerase. The PCR products, P1 (405 pb), P2 (210 pb) and $\beta_2$m (620 pb) were separated on an agarose gel in presence of ethidium bromide and quantified by image analysis.

### Cell cycle distribution analysis

Cell cycle distribution was measured before and after 5-FU exposure. Cell samples for flow cytometry were washed with PBS, resuspended in 0.1% sodium citrate, 0.1% Triton X100 and 50 µg $ml^{-1}$ propidium iodide (PI), and then stored for 24 h at 4°C. After centrifugation at 1500 rpm for 5 min, the samples were resuspended in PBS containing 250 µg $ml^{-1}$ RNAse. Bivariate distributions of cells number vs DNA content (PI) were analysed, using an Orthocyte flow cytometer (Ortho Diagnostic Systems, Roissy, France) equipped with xenon lamp and filter set for excitation at 488 nm. PI fluorescence intensity was recorded through 575 nm high pass filters. At least 20 000 events were collected in each final gated histogram. The data were analysed using Multicycle software (Phoenix Flow Systems, San Diego, CA, USA).

### Western blot analysis and ELISA

Cells were collected in PBS, washed twice, and lysed in ice-cold lysis buffer (20 mmol $l^{-1}$ Tris-HCl pH 8.0, 100 mmol $l^{-1}$ NaCl, 1% Triton X100, 0.5% sodium-deoxycholate, 0.1% SDS, 1 mmol $l^{-1}$sodium-EDTA). Samples were incubated for 30 min on ice then stored at –80°C until analysed. Defrosted samples received 20 µg of protein in Laemmli buffer (Bio Rad) then boiled for 5 min, subjected to SDS-PAGE (10% and 15% respectively for p53 and Bax protein) and were transferred onto Immobilon-P transfer membranes (Millipore, St Quentin Y velynes, France) for p53 or to yuelines Immun-Blot® PVDF membranes (Bio Rad) for Bax, using semi-dry blotting techniques. Membranes were probed for 1 h with DO-7 mouse p53 primary monoclonal antibody or overnight at 4°C with Bax primary polyclonal antibody, and then probed with horseradish peroxidase-labelled secondary antibody for 1 h at room temperature. Immunological complexes were visualized by chemiluminescence detection according to the manufacturer's recommendations (Amersham-Pharmacia Biotech).

Bcl-2 enzyme-linked immunosorbent assay (ELISA) was performed using Amersham-Pharmacia Biotech kit and according to the manufacturer's specifications.

### Statistical analysis

Unless indicated, all data are mean values ± standard deviation (SD) calculated from at least four independent experiments. Spearman's rank correlation was used to test the correlation between the different parameters and Mann and Whitney U test was used to test for the significance level between independent variables.

### RESULTS

### p53 status of cell lines displaying different 5-FU sensitivity

The six carcinoma cell lines displayed a marked difference in 5-FU sensitivity with $IC_{50}$ values ranging from 0.16 ± 0.01 to 22.62 ± 3.09 mM for CAL51 and PANC3 lines, respectively (Table 1). The cell lines were first checked for p53 mutations by direct DNA sequencing. These data are summarized in Table 1. Germline polymorphism at codon 72 in exon 4 (G to C transversion) was detected in CAL51, CAL27 and CAL33 cell lines inducing arginine to proline amino-acid substitution (Matlashewski et al, 1987; Ara et al, 1990). Mutated sequence was

 *© 2000 Cancer Research Campaign*

**Table 2** p53 mRNA expression induction, p53 protein induction after 24 h 5-FU equitoxic exposure

| Cell lines | p53/β2 m RER[a] (%) | p53 protein induction[c] |
|---|---|---|
| CAL51 (wt) | 73 ± 6 | +++ |
| KB (wt) | 238 ± 22 | no |
| FaDu (mt) | 109 ± 9 | ++ |
| CAL33 (mt) | 150 ± 13 | + |
| CAL27 (mt) | 121 ± 23 | + |
| PANC3 (del) | no[b] | no |

[a] mean ± standard deviation of five independent experiments; [b] no RT-PCR mRNA expression; [c]+ = weakly; ++ = moderately; +++ = highly overexpressed; no = no protein expression

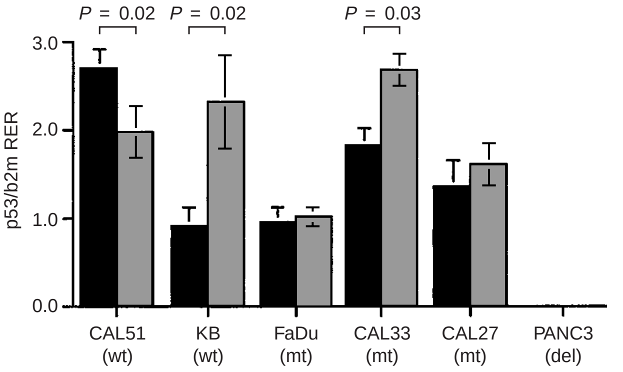

**Figure 1** p53 mRNA relative expression ratio vs β2m before ■ and after ■ 5-FU equitoxic exposure. Results are mean values of five independent experiments

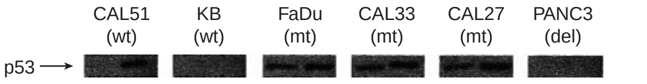

**Figure 2** p53 protein expression before (left) and after (right) 5-FU equitoxic exposure

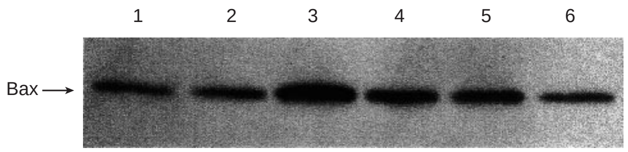

**Figure 3** Bax protein basal expression levels evaluated by western blot analysis. Lanes: 1 = CAL27; 2 = CAL33; 3 = CAL51; 4 = KB; 5 = FaDu; 6 = PANC3

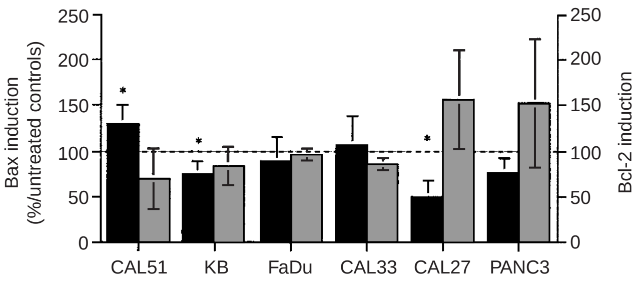

**Figure 4** Bax ■ and Bcl-2 ■ protein expression after 5-FU treatment. Results are mean values of three independent experiments observed in three cell lines. A to T transversion at codon 193 in exon 6 was found in CAL27, resulting in histidine to leucine substitution. Point mutation was detected in CAL33 line (G to A transition) at codon 175 in exon 5, inducing arginine to histidine amino-acid substitution. Point mutation (G to T transversion) at codon 248 in exon 7, resulting in arginine to leucine substitution, was detected in FaDu cells. Internal sequence deletion corresponding to exons 2–4 was evidenced in PANC3 cells. Wild-type p53 status was found in CAL51 and KB cells.

### p53 mRNA and protein expression after 5-FU treatment p53 mRNA and protein expression were determined after cellular stress induced by equitoxic concentrations of 5-FU (Table 2). Figures 1 and 2 show that p53 mRNA RER as well as protein expression were significantly altered after 24-h 5-FU exposure. Mutant p53 cell lines displayed either no modification or an increase of p53 mRNA RER (Figure 1) and p53 protein was slightly up-regulated (Figure 2). p53 mRNA RER was significantly decreased in wild-type p53 CAL51 cell line (Figure 1) and p53 protein was found to be highly overexpressed (Figure 2). Despite p53 wild-type status in KB line, p53 mRNA expression was also found to be up-regulated (Figure 1) and p53 protein was not detected (Figure 2).

### p53 protein functionality as transcription factor: mdm2 and p21 mRNA expression mdm2 mRNA expression before and after 5-FU exposure was reported in Table 3. In the wild-type-p53 CAL51 and KB a significant increase in mdm2 transcription at p53 responsive element (mdm2-p53RE) was observed. No overexpression was detected when p53 was mutated or deleted, except in FaDu cell line which displayed a 1.5-fold increase in mdm2-p53RE (Table 3).

**Table 3** p53 functionality as transcription factor

| | mdm2-p53RE RER | | p21 RER | |
|---|---|---|---|---|
| Cell lines | Control | After 5-FU | Control | after 5-FU |
| CAL51 (wt) | 3.3 ± 1.0 | 6.0 ± 1.8 | 4.0 ± 0.7 | 7.8 ± 1.5 |
| KB (wt) | 0.1 ± 0.0 | 0.5 ± 0.0 | 4.0 ± 0.3 | 5.7 ± 0.4 |
| FaDu (mt) | 0.2 ± 0.0 | 0.3 ± 0.0 | 2.4 ± 0.1 | 3.5 ± 0.1 |
| CAL33 (mt) | 0.1 ± 0.0 | 0.1 ± 0.0 | 2.3 ± 0.2 | 3.5 ± 0.3 |
| CAL27 (mt) | 0.3 ± 0.0 | 0.1 ± 0.0 | 2.8 ± 0.1 | 3.4 ± 0.3 |
| PANC3 (del) | 1.6 ± 0.3 | 0.5 ± 0.1 | 2.8 ± 0.3 | 3.3 ± 0.2 |

p21 mRNA basal expression was higher in CAL51 and KB wt cells. Up-regulation of p21 mRNA was detected in all cell lines, except in PANC3 cell line. Overexpression, however, was higher in CAL51 wt cell lines.

### Cell cycle distribution after 5-FU exposure: p53 status consequences

After 5-FU exposure, all cell lines were able to arrest in G1 phase (Table 4). Nevertheless, only CAL51, KB and FaDu cell lines displayed statistically significant accumulation in G1 phase. S phase was unchanged or slightly decreased and G2/M phase was more markedly reduced (Table 4).

© 2000 Cancer Research Campaign 

**Table 4** Cell cycle distribution after 5-FU exposure (percentage vs untreated controls)

| Cell lines | G1 (%) | S (%) | G2/M (%) |
|---|---|---|---|
| CAL51 (wt) | 130 ± 15 | 89 ± 14 | 27 ± 20 |
| KB (wt) | 120 ± 16 | 102 ± 19 | 25 ± 38 |
| FaDu (mt) | 126 ± 10 | 77 ± 8 | 50 ± 47 |
| CAL33 (mt) | 135 ± 20 | 76 ± 1 | 39 ± 46 |
| CAL27 (mt) | 111 ± 32 | 104 ± 35 | 65 ± 49 |
| PANC3 (del) | 108 ± 8 | 100 ± 12 | 66 ± 33 |

### Bax and Bcl-2 proteins expression after 5-FU exposure: relationship with 5-FU sensitivity and p53 status

As Bax and Bcl-2 proteins are under p53 protein control, changes associated with equitoxic concentration of 5-FU were investigated. After 5-FU exposure, Bcl-2 protein expression decreased in the wild-type p53 cell lines (even in HPV-positive KB cells) whereas in the mutant p53 cell lines no variation (CAL33 and FaDu) or a significant increase (CAL27 and PANC3, Figure 4) was detected. Bcl-2 induction was significantly correlated with 5-FU sensitivity ($r$ = 0.47, $P$ = 0.0323, Figure 5A). Bax was found to be relatively overexpressed in the wild-type p53 CAL51 cell line, but neither in the mutant p53, nor in the HPV-positive cell lines (Figure 3). Moreover, Bax basal levels were related to 5-FU sensitivity, since the most sensitive cell line (CAL51) displayed the highest Bax level as opposed to data achieved in the most resistant cell line (PANC3, Figure 3). Bax induction was significantly correlated with 5-FU sensitivity ($r$ = 0.65, $P$ = 0.0054, Figure 5B). Bcl-2/Bax proteins ratio was also correlated with 5-FU sensitivity ($r$ = 0.88, $P$ = 0.0097, Figure 5C).

### DISCUSSION

Among the six human cancer cell lines selected and exhibiting a wide range of sensitivity to 5-FU, CAL51 and KB cell lines displayed wild-type p53 profile: wild-type gene and undetectable basal protein expression, three out of six cell lines showed point mutations of p53 gene and constitutive p53 protein expression. PANC3 cell line displayed internal gene deletion resulting in complete lack of p53 mRNA and protein expression. Wt p53 cell lines were more sensitive to 5-FU than mutated lines. Our results support the concept that cells carrying wt gene tend to be sensitive to 5-FU and that deletion of p53 function results in resistance. In the present experiments, 5-FU induced an increase in p53 mRNA expression in mutant-type cell lines and in HPV-positive wild-type cell lines, whereas in CAL51 wild-type p53 cell line, a significant decrease in p53 gene expression was observed (0.7-fold, $P$ = 0.02). These results are in agreement with those reported by Palmer et al (1997). Cellular mechanisms able to regulate wt p53 function include post-translational stabilization (Kastan et al, 1991), nuclear exclusion or cytoplasmic sequestration (Moll et al, 1996), negative feedback inhibition of p53 mRNA translation by p53 protein itself (Mosner et al, 1995), binding of p53 by proteins such as mdm2 (Momand et al, 1992) or HPV E6 (Crook et al, 1991). Moreover, regulation of p53 protein could also implicate the changes in the p53 gene transcription such as p53 mRNA half-life modification or CpG nucleotides methylation (Kren et al, 1996). Conversely, regulation of mutated p53 levels after drug treatment consisted in an increase in translation process (Nabeya et al, 1995). In the present study, 5-FU exposure was found to induce an increase in p53 mRNA and protein expression in mutated cell lines. As currently accepted, biosynthesis of wild-type p53 can be controlled by both transcriptional (Deffie et al, 1993; Hudson et al, 1995) and translational (Mosner et al, 1995; Ewen and Miller, 1996; Fu et al, 1996) regulation processes. Our results are consistent with Nabeya et al (1995), demonstrating that an increase in wild-type p53 protein levels was mainly due to post-translational stabilization. Nevertheless, despite p53 wild-type status in KB line, p53 protein was not up-regulated and remained undetectable after exposure to 5-FU. In fact, KB cell line was described as containing HPV-18 sequences (Boshart et al, 1984). HPV E6 protein was shown to actively stimulate the degradation of bound p53 through ubiquitin-dependent proteolysis (Scheffner et al, 1990; Crook et al, 1991; Huibregtse et al, 1993) and HPV E7 protein could also inhibit p53 transcriptional activity by binding p53 in presence of TATA box-binding protein (Massimi and Banks, 1997). Consequently, cell lines containing HPV-16 and HPV-18 oncogenic human papilloma virus should not display any up-regulation of p53 protein despite a wild-type status.

Whether p53 protein up-regulation observed could correspond to p53 transcriptional ability was tested through the induction of mdm2 and p21 transactivation. Mdm2 gene possesses a p53-responsive element (mdm2-p53RE) (Barak et al, 1994; Zauberman

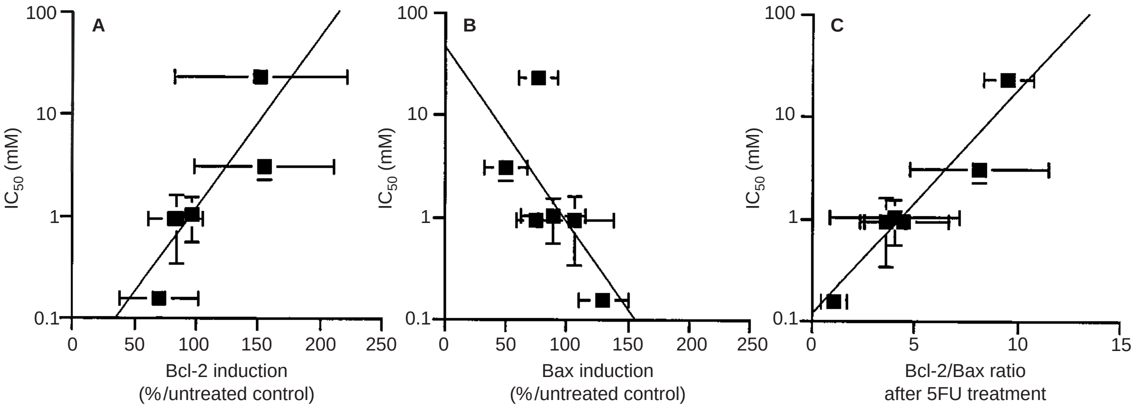

**Figure 5** Relationship between 5-FU sensitivity and (**A**) Bcl-2 protein induction ($r$ = 0.47, $P$ = 0.0323), (**B**) bax protein induction ($r$ = 0.65, $P$ = 0.0054) and (**C**) between Bcl-2/Bax protein ratio ($r$ = 0.88, $P$ = 0.0097), after exposure to 5-FU

*© 2000 Cancer Research Campaign*

et al, 1995), moreover p53 and mdm2 proteins constitute an autoregulatory feedback loop in which p53 activity is self-limited through mdm2 production (Kubbutat et al, 1997). Wild-type cell lines (CAL51 and KB) showed a marked increase in mdm2 transcript corresponding to mdm2-p53RE and no up-regulation was detected when p53 gene was mutated or deleted, except for FaDu. Mutant p53 was previously demonstrated to be able to bind mdm2-p53RE, according to the nature and position of the amino-acid substitution (Gorgoulis et al, 1998). O'Connor et al (1993) also demonstrated induction of mdm2 mRNA for three mutant p53 lines, which exhibited alteration of codon 248 as observed in FaDu cell line. This mutation was classified as 'contact mutant', since residue 248 is directly involved in p53 DNA-binding, and then reduced the affinity of p53 for its consensus sites by removing critical contact with DNA (Cho et al, 1994; Arrowsmith and Morin, 1996) without changing p53 conformation (Ory et al, 1994). p21 mRNA basal expression was reduced in p53 mutated cell lines in agreement with Elbendary et al (1996). Relationship could be evidenced between p21 basal expression and p53 status. p21 mRNA overexpression was still detected in all cell lines independently of p53 status, suggesting that p21 could be up-regulated by other pathways (Macleod et al, 1995; Loignon et al, 1997; Wouters et al, 1999). In all cell lines after 5-FU exposure p21 induction, mediated or not by p53, results in G1 arrest (el-Deiry et al, 1993; 1994). However, G1 accumulation was statistically significant only for CAL51, KB, and FaDu lines, which exhibited p53 transcriptional functionality leading to G1 arrest, which should be more related to p53 functionality than p53 status.

Since Bax was identified as a p53 early-response gene (Selvakumaran et al, 1994), and a unique p53-regulated gene which induced apoptosis (Zhan et al, 1994), and Bcl-2 as an apoptosis antagonist (Oltvai et al, 1993; Reed, 1994), levels of Bax and Bcl-2 were analysed in cell lines after exposure to 5-FU equitoxic concentrations. Bcl-2 family proteins were demonstrated to be important apoptosis regulators after 5-FU treatment (Koshiji et al, 1997; Nita et al, 1998*a*). Likewise, CAL51 cell line displayed a significant increase in Bax, as well as a significant decrease in Bcl-2 protein expression. All p53 mutant cell lines displayed either no modification of Bax and Bcl-2 protein expression (CAL33 and FaDu), or a significant decrease in Bax as well as an increase in Bcl-2 protein expression (CAL27 and PANC3) after 5-FU exposure. Our results also showed that HPV-18 positive KB cell line, in which p53 was not up-regulated, displayed no Bax protein up-regulation, but Bcl-2 down regulation. 5-FU-resistant cell lines (CAL27 et PANC3) showed an increase in Bcl-2 protein expression reported to protect the cells against thymidylate synthase inhibitor (Fisher et al, 1993), as well as other anticancer drugs (Reed, 1994). Bcl-2 and Bax induction significantly correlates with 5-FU sensitivity and whatever p53 status, Bcl-2 to Bax relative expression ratio was also correlated with 5-FU sensitivity.

In vitro, 5-FU sensitivity was related to different mechanisms (Pinedo and Peters, 1988; Spears et al, 1988; Zhang et al, 1992; Peters et al, 1995). Although each of the mechanisms have been well documented, their relative contribution to the development of clinical drug resistance remains incertain. However, there is a growing body of evidence to suggest that sensitivity to the cytotoxic effects of fluoropyrimidines may be mediated via TS and DPD process (Peters et al, 1995). Although TS and DPD have demonstrated potential prognostic significance, their prognostic values are still controversial (Beck et al, 1994; Nita et al, 1998*b*; Etienne et al, 1999; Kirihara et al, 1999). More recently, Bcl-2 family proteins were implicated in chemotherapy-induced cell death (Simonian et al, 1997) and previous results suggest that some members of the Bcl-2 family of proteins, in human colon cancer cell lines, are modulated by 5-FU, and that the ratio of Bcl-X(L) to Bax may be related to chemosensitivity to 5-FU (Nita et al, 1998*a*).

In conclusion, for cell cycle control, p53 functionality appeared to be more essential than mutational status. Moreover, whatever p53 status or functionality, 5-FU sensitivity was related to Bcl-2 family proteins expression and Bcl-2/Bax ratio could be a relevant marker to predict 5-FU treatment response.

### ACKNOWLEDGEMENTS

This work was performed within the framework of the 'Pôle Européen de Santé Région Lorraine Communauté Urbaine du Grand Nancy' and was supported by the French 'Ligue contre le Cancer'.

### REFERENCES

Aguilar Santelises M, Rottenberg ME, Lewin N, Mellstedt H and Jondal M (1996) Bcl-2, Bax and p53 expression in B-CLL in relation to in vitro survival and clinical progression. *Int J Cancer* **69**: 114–119

Ara S, Lee PS, Hansen MF and Saya H (1990) Codon 72 polymorphism of the TP53 gene. *Nucleic Acids Res* **18**: 4961

Arrowsmith CH and Morin P (1996) New insights into p53 function from structural studies. *Oncogene* **12**: 1379–1385

Barak Y, Juven T, Haffner R and Oren M (1993) mdm2 expression is induced by wild type p53 activity. *EMBO J* **12**: 461–468

Barak Y, Gottlieb E, Juven Gershon T and Oren M (1994) Regulation of mdm2 expression by p53: alternative promoters produce transcripts with nonidentical translation potential. *Genes Dev* **8**: 1739–1749

Barberi-Heyob M, Griffon G, Merlin JL and Weber B (1993) Sequence-dependent growth-inhibitory effects of the in vitro combination of fluorouracil, cisplatin and dipyridamole. *Cancer Chemother Pharmacol* **33**: 163–170

Beck A, Etienne MC, Cheradame S, Fischel JL, Formento P, Renee N and Milano G (1994) A role for dihydropyrimidine dehydrogenase and thymidylate synthase in tumour sensitivity to fluorouracil. *Eur J Cancer* **30a**: 1517–1522

Boshart M, Gissmann L, Ikenberg H, Kleinheinz A, Scheurlen W and zur Hausen H (1984) A new type of papillomavirus DNA, its presence in genital cancer biopsies and in cell lines derived from cervical cancer. *EMBO J* **3**: 1151–1157

Cho Y, Gorina S, Jeffrey PD and Pavletich NP (1994) Crystal structure of a p53 tumor suppressor – DNA complex: understanding tumorigenic mutations [see comments]. *Science* **265**: 346–355

Chou TC and Talalay P (1987) Application of the median-effect principle for the assessment of low dose risk of carcinogens and for the quantitation of synergism and antagonism of chemotherapeutic agents. In *New Avenues in Developmental Cancer Chemotherapy*, Harrap KR and Connors TA (eds) pp. 37–64. Academic Press: New York

Crook T, Tidy JA and Vousden KH (1991) Degradation of p53 can be targeted by HPV E6 sequences distinct from those required for p53 binding and trans-activation. *Cell* **67**: 547–556

Deffie A, Wu H, Reinke V and Lozano G (1993) The tumor suppressor p53 regulates its own transcription. *Mol Cell Biol* **13**: 3415–3423

Dou QP, An B and Will PL (1995) Induction of a retinoblastoma phosphatase activity by anticancer drugs accompanies p53-independent G1 arrest and apoptosis. *Proc Natl Acad Sci USA* **92**: 9019–9023

Elbendary AA, Cirisano FD, Evans AC, Jr., Davis PL, Iglehart JD, Marks JR and Berchuck A (1996) Relationship between p21 expression and mutation of the p53 tumor suppressor gene in normal and malignant ovarian epithelial cells. *Clin Cancer Res* **2**: 1571–1575

el-Deiry WS, Tokino T, Velculescu VE, Levy DB, Parsons R, Trent JM, Lin D, Mercer WE, Kinzler KW and Vogelstein B (1993) WAF1, a potential mediator of p53 tumor suppression. *Cell* **75**: 817–825

el-Deiry WS, Harper JW, O'Connor PM, Velculescu VE, Canman CE, Jackman J, Pietenpol JA, Burrell M, Hill DE and Wang Y (1994) WAF1/CIP1 is induced in p53-mediated G1 arrest and apoptosis. *Cancer Res* **54**: 1169–1174

© 2000 Cancer Research Campaign

Etienne MC, Pivot X, Formento JL, Bensadoun RJ, Formento P, Dassonville O, Francoual M, Poissonnet G, Fontana X, Schneider M, Demard F and Milano G (1999) A multifactorial approach including tumoural epidermal growth factor receptor, p53, thymidylate synthase and dihydropyrimidine dehydrogenase to predict treatment outcome in head and neck cancer patients receiving 5-fluorouracil. *Br J Cancer* **79**: 1864–1869

Ewen ME and Miller SJ (1996) p53 and translational control. *Biochim Biophys Acta* **1242**: 181–184

Fisher TC, Milner AE, Gregory CD, Jackman AL, Aherne GW, Hartley JA, Dive C and Hickman JA (1993) bcl-2 modulation of apoptosis induced by anticancer drugs: resistance to thymidylate stress is independent of classical resistance pathways. *Cancer-Res* **53**: 3321–3326

Fu L, Minden MD and Benchimol S (1996) Translational regulation of human p53 gene expression. *EMBO J* **15**: 4392–4401

Gorgoulis VG, Zacharatos PV, Manolis E, Ikonomopoulos JA, Damalas A, Lamprinopoulos C, Rassidakis GZ, Zoumpourlis V, Kotsinas A, Rassidakis AN, Halazonetis TD and Kittas C (1998) Effects of p53 mutants derived from lung carcinomas on the p53-responsive element (p53RE) of the MDM2 gene. *Br J Cancer* **77**: 374–384

Guillouf C, Grana X, Selvakumaran M, De Luca A, Giordano A, Hoffman B and Liebermann DA (1995) Dissection of the genetic programs of p53-mediated G1 growth arrest and apoptosis: blocking p53-induced apoptosis unmasks G1 arrest. *Blood* **85**: 2691–2698

Gussow D, Rein R, Ginjaar I, Hochstenbach F, Seemann G, Kottman A and Ploegh HL (1987) The human beta 2-microglobulin gene. Primary structure and definition of the transcriptional unit. *J Immunol* **139**: 3132–3138

Harris CC (1996) Structure and function of the p53 tumor suppressor gene: clues for rational cancer therapeutic strategies. *J Natl Cancer Inst* **88**: 1442–1455

Hudson JM, Frade R and Bar Eli M (1995) Wild-type p53 regulates its own transcription in a cell-type specific manner. *DNA Cell Biol* **14**: 759–766

Huibregtse JM, Scheffner M and Howley PM (1993) Cloning and expression of the cDNA for E6-AP, a protein that mediates the interaction of the human papillomavirus E6 oncoprotein with p53. *Mol Cell Biol* **13**: 775–784

Kastan MB, Onyekwere O, Sidransky D, Vogelstein B and Craig RW (1991) Participation of p53 protein in the cellular response to DNA damage. *Cancer Res* **51**: 6304–6311

Kirihara Y, Yamamoto W, Toge T and Nishiyama M (1999) Dihydropyrimidine dehydrogenase, multidrug resistance-associated protein, and thymidylate synthase gene expression levels can predict 5-fluorouracil resistance in human gastrointestinal cancer cells. *Int J Oncol* **14**: 551–556

Koshiji M, Adachi Y, Taketani S, Takeuchi K, Hioki K and Ikehara S (1997) Mechanisms underlying apoptosis induced by combination of 5-fluorouracil and interferon-gamma. *Biochem Biophys Res Commun* **240**: 376–381.

Kren BT, Trembley JH and Steer CJ (1996) Alterations in mRNA stability during rat liver regeneration. *Am J Physiol* **270**: G763–777

Kubbutat MH, Jones SN and Vousden KH (1997) Regulation of p53 stability by Mdm2. *Nature* **387**: 299–303

Kuerbitz SJ, Plunkett BS, Walsh WV and Kastan MB (1992) Wild-type p53 is a cell cycle checkpoint determinant following irradiation. *Proc Natl Acad Sci USA* **89**: 7491–7495

Loignon M, Fetni R, Gordon AJ and Drobetsky EA (1997) A p53-independent pathway for induction of p21 waflcip1 and concomitant G1 arrest in UV-irradiated human skin fibroblasts. *Cancer Res* **57**: 3390–3394

Lowe SW, Ruley HE, Jacks T and Housman DE (1993) p53-dependent apoptosis modulates the cytotoxicity of anticancer agents. *Cell* **74**: 957–967

Macleod KF, Sherry N, Hannon G, Beach D, Tokino T, Kinzler K, Vogelstein B and Jacks T (1995) p53-dependent and independent expression of p21 during cell growth, differentiation, and DNA damage. *Genes Dev* **9**: 935–944

Massimi P and Banks L (1997) Repression of p53 transcriptional activity by the HPV E7 proteins. *Virology* **227**: 255–259

Matlashewski GJ, Tuck S, Pim D, Lamb P, Schneider J and Crawford LV (1987) Primary structure polymorphism at amino acid residue 72 of human p53. *Mol Cell Biol* **7**: 961–963

Moll UM, Ostermeyer AG, Haladay R, Winkfield B, Frazier M and Zambetti G (1996) Cytoplasmic sequestration of wild-type p53 protein impairs the G1 checkpoint after DNA damage. *Mol Cell Biol* **16**: 1126–1137

Momand J, Zambetti GP, Olson DC, George D and Levine AJ (1992) The mdm-2 oncogene product forms a complex with the p53 protein and inhibits p53-mediated transactivation. *Cell* **69**: 1237–1245

Mosner J, Mummenbrauer T, Bauer C, Sczakiel G, Grosse F and Deppert W (1995) Negative feedback regulation of wild-type p53 biosynthesis. *EMBO J* **14**: 4442–4449

Nabeya Y, Loganzo F, Jr., Maslak P, Lai L, de Oliveira AR, Schwartz GK, Blundell ML, Altorki NK, Kelsen DP and Albino AP (1995) The mutational status of p53 protein in gastric and esophageal adenocarcinoma cell lines predicts sensitivity to chemotherapeutic agents. *Int J Cancer* **64**: 37–46

Nita ME, Nagawa H, Tominaga O, Tsuno N, Fujii S, Sasaki S, Fu CG, Takenoue T, Tsuruo T and Muto T (1998*a*) 5-Fluorouracil induces apoptosis in human colon cancer cell lines with modulation of Bcl-2 family proteins. *Br J Cancer* **78**: 986–992

Nita ME, Tominaga O, Nagawa H, Tsuruo T and Muto T (1998*b*) Dihydropyrimidine dehydrogenase but not thymidylate synthase expression is associated with resistance to 5-fluorouracil in colorectal cancer. *Hepatogastroenterology* **45**: 2117–2122

O’Connor PM, Jackman J, Jondle D, Bhatia K, Magrath I and Kohn KW (1993) Role of the p53 tumor suppressor gene in cell cycle arrest and radiosensitivity of Burkitt’s lymphoma cell lines. *Cancer Res* **53**: 4776–4780

Oltvai ZN, Milliman CL and Korsmeyer SJ (1993) Bcl-2 heterodimerizes in vivo with a conserved homolog, Bax, that accelerates programmed cell death. *Cell* **74**: 609–619

Oren M (1994) Relationship of p53 to the control of apoptotic cell death. *Semin Cancer Biol* **5**: 221–227

Ory K, Legros Y, Auguin C and Soussi T (1994) Analysis of the most representative tumour-derived p53 mutants reveals that changes in protein conformation are not correlated with loss of transactivation or inhibition of cell proliferation. *EMBO J* **13**: 3496–3504

Palmer DG, Paraskeva C and Williams AC (1997) Modulation of p53 expression in cultured colonic adenoma cell lines by the naturally occurring lumenal factors butyrate and deoxycholate. *Int J Cancer* **73**: 702–706

Peters GJ, van der Wilt CL, van Triest B, Codacci-Pisanelli G, Johnston PG, van Groeningen CJ and Pinedo HM (1995) Thymidylate synthase and drug resistance. *Eur J Cancer* **31A**: 1299–1305

Pinedo HM and Peters GF (1988) Fluorouracil: biochemistry and pharmacology. *J Clin Oncol* **6**: 1653–1664

Reed JC (1994) Bcl-2 and the regulation of programmed cell death. *J Cell Biol* **124**: 1–6

Scheffner M, Werness BA, Huibregtse JM, Levine AJ and Howley PM (1990) The E6 oncoprotein encoded by human papillomavirus types 16 and 18 promotes the degradation of p53. *Cell* **63**: 1129–1136

Selvakumaran M, Lin HK, Miyashita T, Wang HG, Krajewski S, Reed JC, Hoffman B and Liebermann D (1994) Immediate early up-regulation of bax expression by p53 but not TGF beta 1: a paradigm for distinct apoptotic pathways. *Oncogene* **9**: 1791–1798

Simonian PL, Grillot DA and Nunez G (1997) Bcl-2 and Bcl-XL can differentially block chemotherapy-induced cell death. *Blood* **90**: 1208–1216

Spears CP, Gustavsson BG, Berne M, Frösing R, Bernstein L and Hayes AA (1988) Mechanisms of innate resistance to thymidylate synthase inhibition after 5-fluorouracil. *Cancer Res* **48**: 5894–5900

Stewart N, Hicks GG, Paraskevas F and Mowat M (1995) Evidence for a second cell cycle block at G2/M by p53. *Oncogene* **10**: 109–115

Strobel T, Swanson L, Korsmeyer S and Cannistra SA (1996). BAX enhances paclitaxel-induced apoptosis through a p53-independent pathway. *Proc Natl Acad Sci USA* **93**: 14094–14099

Vogelstein B and Kinzler KW (1992). p53 function and dysfunction. *Cell* **70**: 523–526

Wouters BG, Denko NC, Giaccia AJ and Brown JM (1999). A p53 and apoptotic-independent role for p21 waf1 in tumour response to radiation therapy. *Oncogene* **18**: 6540–6545

Zauberman A, Flusberg D, Haupt Y, Barak Y and Oren M (1995). A functional p53-responsive intronic promoter is contained within the human mdm2 gene. *Nucleic Acids Res* **23**: 2584–2592

Zhan Q, Fan S, Bae I, Guillouf C, Liebermann DA, O’Connor PM and Fornace AJ, Jr. (1994). Induction of bax by genotoxic stress in human cells correlates with normal p53 status and apoptosis (published erratum appears in *Oncogene* 1995; **10**(6): 1259) *Oncogene* **9**: 3743–3751

Zhang ZG, Harstrick A and Rustum YM (1992) Mechanisms of resistance to fluoropyrimidines. *Semin Oncol* **19**: 4–9

 *© 2000 Cancer Research Campaign*
